# Monte Carlo simulation of neutron scattering by a textured polycrystal

**DOI:** 10.1107/S1600576720002290

**Published:** 2020-03-30

**Authors:** Victor Laliena, Miguel Ángel Vicente-Álvarez, Javier Campo

**Affiliations:** a Instituto de Ciencia de Materiales de Aragón (CSIC – Universidad de Zaragoza) and Departamento de Física de Materia Condensada, Universidad de Zaragoza, C/Pedro Cerbuna 12, E-50009 Zaragoza, Spain; b Departamento Física de Neutrones, LAHN project, Centro Atómico Bariloche, CNEA/CONICET, SC de Bariloche, Argentina

**Keywords:** neutron diffraction, crystallographic texture, Monte Carlo simulations

## Abstract

A method of simulating the neutron scattering by a textured polycrystal is presented. It is based on an expansion of the scattering cross sections in terms of the spherical harmonics of the incident and scattering directions, which is derived from the generalized Fourier expansion of the polycrystal orientation distribution function. The method has been implemented in a Monte Carlo code as a component of the *McStas* software package, and it has been validated by computing some pole figures of a Zircaloy-4 plate and a Zr–2.5Nb pressure tube, and by simulating an ideal transmission experiment. The code can be used to estimate the background generated by components of neutron instruments such as pressure cells, whose walls are made of alloys with significant crystallographic texture. As a first application, the effect of texture on the signal-to-noise ratio was studied in a simple model of a diffraction experiment, in which a sample is placed inside a pressure cell made of a zirconium alloy. With this setting, the results of two simulations were compared: one in which the pressure-cell wall has a uniform distribution of grain orientations, and another in which the pressure cell has the texture of a Zr–2.5Nb pressure tube. The results showed that the effect of the texture of the pressure cell on the noise of a diffractogram is very important. Thus, the signal-to-noise ratio can be controlled by appropriate choice of the texture of the pressure-cell walls.

## Abstract 2

A method of simulating the diffraction of thermal neutrons by polycrystalline materials, using the generalized Fourier transform of the orientation distribution function, is developed and implemented in a Monte Carlo code in the *McStas* software package. Simulations to validate the code and a first application are also presented.

## 1. Introduction   

Neutron and X-ray scattering are extensively used in materials science for many purposes, in particular to analyse the structure of phases, quantifying their volume fractions and determining the state of stress and the crystallographic texture. The continuous demand for these techniques by the technological and scientific community gave rise to the construction of dedicated instruments at neutron and synchrotron facilities. Because of the low flux of neutrons compared with X-rays, in neutron laboratories the instruments are optimized for a particular set of scientific applications, which implies looking for the highest flux on the sample while keeping the resolution required by the technique to ensure a reasonable signal-to-noise ratio (SNR). The experimental setup determined by the optimization defines the characteristics of the beam impinging on the sample, which in turn influences the measurements, for instance the shape and position of the diffraction peaks (Mikula *et al.*, 1997 ▸; Stoica *et al.*, 2001 ▸).

Knowing how the instrument configuration affects the measurements is important not only during the design process of the instrument but also during operation, to interpret the bias of the experimental observations. The large number of variables that define the instrument configuration gives rise to an increasing use of Monte Carlo simulations. In these models, the neutron travels from the source to the detector and in its path interacts with the different components of the instrument and, eventually, with the sample. Examples of such software are the *McStas* package (Lefmann & Nielsen, 1999 ▸), the *VITESS* project (Zsigmond *et al.*, 2002 ▸), *IB* (Zhao, 2011 ▸) and *IDEAS* (Lee & Wang, 2002 ▸), among others. Monte Carlo engines have also been added to some analysis programs like *RESTRAX* (Šaroun & Kulda, 1997 ▸) and have been used to estimate the corrections needed to extract physical quantities from experimental measurements (Vickery *et al.*, 2013 ▸). A good example is the estimation of pseudo-stresses in neutron diffraction experiments (Šaroun *et al.*, 2013 ▸). Attempts at realistic simulations by combining detailed instrument and sample modelling were presented by Farhi *et al.* (2009 ▸) and by Lin *et al.* (2016 ▸).

Monte Carlo simulations are particularly important for neutron instruments due to the large gauge volume necessary to have a significant signal, the reason being the low brightness of neutron sources compared with synchrotron or even laboratory X-ray instruments. This large volume brings about unwanted spatial resolution effects called pseudo-strains, which are caused by perturbation of the instrumental gauge volume due to the heterogeneous distribution of the scattering probability in the sample. The surface effect when the gauge volume is only partially immersed in the material is a well known special case. In general, any heterogeneity or beam extinction mechanism which causes significant variation of the scattering probability on a distance comparable to the gauge size may give rise to pseudo-strains, for example gradients in phase composition and texture, or a strong variation in beam attenuation with wavelength near a Bragg edge. The pseudo-strains are often of the same magnitude as the measured lattice strain and need to be properly treated. Monte Carlo models proved to be useful for this objective since they can account for beam attenuation, multiple scattering, divergence effects *etc*.

Another important application of Monte Carlo modelling is related to estimation of the SNR. In some cases, for example when using sample environment devices like furnaces or pressure cells, the neutron travels through the device wall before reaching the sample and/or the detector. In its path, it may suffer multiple scattering, either elastic or inelastic, increasing the instrument background. This is particularly important in high-pressure neutron instruments, where the pressure cell has a thick wall (Rodríguez-Velamazán *et al.*, 2011 ▸; Rodríguez-Velamazán & Noguera, 2011 ▸). To reduce the background as much as possible, the selection of materials and their fabrication processes are critical. Alloys that minimize the background, such as TiAlV or CuBe, have been proposed (Kibble *et al.*, 2019 ▸). However, to lower the background further, the crystallographic texture can be considered a design variable.

The scattering of neutrons by textured polycrystals, including a detailed description of texture, has not yet been fully incorporated into the available Monte Carlo programs. The *nxs* library to compute the neutron total scattering cross sections (Boin, 2012 ▸), which uses the March–Dollase model (Dollase, 1986 ▸) to include the effect of preferred grain orientations in the amplitude of Bragg edges, was implemented in *McStas* Release 2.5. Concerning the analysis of transmission (Bragg edge) spectra in textured materials, the total coherent cross sections have been implemented in terms of integration of the pole figures (Santisteban *et al.*, 2012 ▸; Malamud *et al.*, 2014 ▸), but these are not suitable for implementation in an efficient Monte Carlo code due to the demanding computational cost. Other tools for analysing transmission data through polycrystalline samples which implement approximations to the total cross section are *Sinpol* (Dessieux *et al.*, 2018 ▸, 2019 ▸) and *RITS* (Sato *et al.*, 2011 ▸), and earlier work includes that of Vogel (1999 ▸).

In this work, we present expressions for the differential and total elastic coherent cross sections in terms of the generalized Fourier coefficients of the orientation distribution function (ODF), which are suitable for implementation in Monte Carlo programs. A closed expression for the total cross section derived here allows a time-efficient evaluation of this quantity, a necessary condition for its use in Monte Carlo simulations. As mentioned above, other expressions for this quantity were obtained earlier (Santisteban *et al.*, 2006 ▸, 2012 ▸). In our case, the truncation of the generalized Fourier series of the ODF renders the Monte Carlo simulations feasible, although they are computationally much more expensive than the standard simulations with single crystals or powder materials. The efficiency can be greatly improved by using variance reduction techniques. These developments have been implemented in a Monte Carlo code as a new component of the *McStas* package.

The paper is organized as follows: in Section 2 we give a brief description of the ODF, to state clearly the conventions used in this work; in Sections 3 and 4 we present, respectively, the expressions used to compute the differential and total neutron cross sections for coherent elastic scattering by a polycrystalline material; in Section 5 we describe in detail how the method is implemented in the *McStas* Monte Carlo code; in Section 6 we analyse the effects of the truncation of the Fourier series; in Section 7 we present the results of simulations performed to validate the code; and in Section 8 we discuss, as a first application, an estimation of the SNR of an experiment involving a pressure cell with a sharp texture, comparing it with the SNR associated with a pressure cell of the same characteristics and a uniform texture. Finally, the methods and results are summarized in Section 9. Some details of the computations and other useful information are provided in the appendices.

## 2. Orientation distribution function   

The crystallographic texture of a polycrystalline sample is characterized by its ODF, which gives the relative number of crystal grains that have a particular orientation. The neutron scattering cross section can be computed from the ODF, under some approximations to be discussed in the next section. Let us recall here the basic properties of the ODF, which serves also to fix the notation.

Let 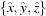 be a right-handed orthonormal triad defining a reference frame attached to the sample, and {**a**, **b**, **c**} a system of three independent crystal lattice vectors that generate the whole lattice, oriented in some fixed specified way with respect to the sample frame. The vectors, **G**, of the reciprocal lattice are determined by the Miller indices *hkl* through 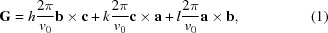
where *v*_0_ = **a** · (**b** × **c**) is the volume of the crystal unit cell.

A polycrystal is a material composed of crystalline grains with different orientations at different sample points. The orientation of a grain at a point **r** in the sample is described by a rotation *g*(**r**) ∈ SO(3) (the three-dimensional rotation group), so that the crystal orientation at a point **r** is given by the triad 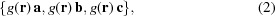
and the vectors of the corresponding reciprocal lattice are given by *g*(**r**)**G**.

The ODF of the polycrystal is a real function 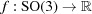 that gives the volume fraction of grains having an orientation with respect to the sample determined by the rotation *g* (Bunge, 1993 ▸). The ODF satisfies *f*(*g*) ≥ 0 and is normalized so that 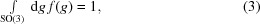
where d*g* is the Haar (invariant) measure on SO(3), normalized so that 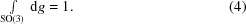
A rotation *g* can be expressed in terms of the three Euler angles (α, β, γ) as 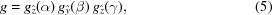
where 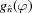 denotes the rotation by an angle φ about the 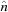 axis. Note that the Euler angles are defined here in terms of rotations about the fixed sample axes 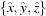, and α and γ take values in [0, 2π] and β in [0, π]. In terms of the Euler angles, the invariant measure has the form 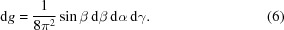



The ODF is the key point of the present work, as it uniquely determines the neutron scattering cross sections in a polycrystalline material, within reasonable assumptions (see next section). In neutron and X-ray diffraction experiments, the ODF is not directly measurable and has to be computed from measurements of related quantities like pole figures. The mathematical problem of extracting the ODF from pole figure measurements is called the pole figure inversion problem and was first addressed in the pioneering work of Bunge (1965 ▸) and Roe (1965 ▸). Since then, several methods have been proposed and perfected by several authors (Pospiech & Jura, 1974 ▸; Jura *et al.*, 1974 ▸, 1976 ▸; Matthies & Pospiech, 1980 ▸; Pospiech *et al.*, 1981 ▸; Houtte, 1983 ▸; Imhof, 1983 ▸; Pawlik, 1986 ▸; Schaeben, 1988 ▸; Matthies, 1988 ▸; Helming & Eschner, 1990 ▸; Houtte, 1991 ▸; Vadon & Heizmann, 1991 ▸; van den Boogaart *et al.*, 2007 ▸; Bernier *et al.*, 2006 ▸; Hielscher & Schaeben, 2008 ▸).

The ODF can be expanded in a generalized Fourier series as (Bunge, 1993 ▸) 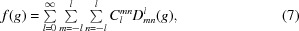
where 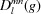 are the Wigner *D* matrices and 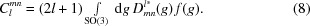
The star superscript stands for complex conjugation. For conciseness, here we call 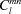 the Fourier coefficients and equation (7) the Fourier series of the ODF, although it is an abuse of language. The relation 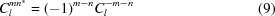
holds by virtue of the reality of the ODF. In terms of the Euler angles, the Wigner matrices are given by 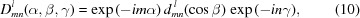
where 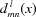 are the Wigner *d* functions, an explicit expression of which is given in Appendix *A* [App appa]. Given an ODF measured on a discrete mesh of SO(3), its Fourier coefficients can be computed with texture analysis software, such as *MTEX* (Hielscher & Schaeben, 2008 ▸).

The Fourier expansion of the ODF is currently used in some Rietveld refinement programs that deal with crystallographic texture, for instance *MAUD* (Lutterotti *et al.*, 1997 ▸, 1999 ▸; Wenk *et al.*, 2010 ▸).

## 3. Neutron scattering differential cross section   

Let us obtain the coherent elastic scattering differential cross section of a neutron propagating through a polycrystalline material. We use the following notation: *N*_c_ is the number of unit cells in a crystal, *v*_0_ the volume of the unit cell, **G** a reciprocal-lattice vector attached to the fixed crystal frame {**a**, **b**, **c**} and *F*_**G**_ the corresponding structure factor. The wavevectors of the incident and scattered neutrons are **k** and **k**′, respectively, and the scattering vector is **q** = **k** − **k**′. We deal only with elastic scattering, so that **k**′ = **k**.

The coherent elastic differential cross section for the scattering by a perfect single crystal, small enough that the kinematical approximation (disregarding primary extinction) holds, is given by (Squires, 1996 ▸) 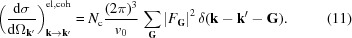
In a polycrystal there is no interference between the scattering produced by different grains, since they are very large in comparison with the neutron wavelength and highly dis­oriented, and thus the cross section is merely the sum of the cross sections due to the individual grains (Sears, 1989 ▸). Furthermore, the grains can be considered as perfect single crystals, since the effect of mosaicity is completely masked by the effect of orientation disorder and can in principle be neglected. Taking all this into account, and given that the number of unit cells with orientation determined by *g* is *N*_c_ *f*(*g*)d*g*, the cross section can be written as 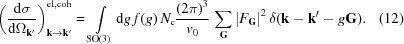
The integral over *g* can be performed before the sum over **G**, and thus we have to compute 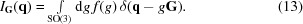
An explicit expression for *I*_**G**_(**q**) can be obtained by using the Fourier expansion of the ODF given by equation (7). Details of the computations are given in Appendix *B* [App appb]. The result is the following expression for the differential cross section: 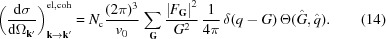
Here, 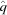 = **q**/*q* is the unit vector along the scattering vector direction, 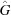 = **G**/*G* and 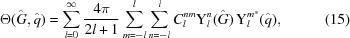
with 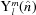 being the spherical harmonics evaluated at the point 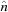 on the unit sphere. Some properties of the spherical harmonics and the conventions used in this work are summarized in Appendix *A* [App appa].

For a uniform ODF (a ‘powder’) 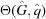 = 1, since the only non-vanishing Fourier coefficient is 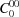 = 1, and the well known expression for the scattering by a powder is recovered (Sears, 1989 ▸). Note that 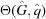 is proportional to the corresponding pole function (Bunge, 1993 ▸): they differ only by the 4π factor entering equation (15). We prefer this normalization because in this way the 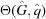 factor is the modulation in 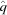 of the powder scattering cross section originating from the texture.

Formulas (14) and (15) for the differential scattering cross section were used previously in models for Rietveld refinement programs for textured polycrystals (Popa, 1992 ▸).

## 4. Total cross section   

The total elastic coherent cross section is obtained by integrating the differential cross section over the direction of the scattered neutron, 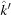: 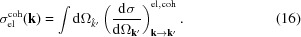
For a textured polycrystal the differential cross section is given by (12). Hence, equation (16) actually involves a double integration over d*g* and 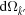. To get an explicit expression in terms of the Fourier coefficients of the ODF we find it convenient to perform the integral over 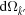 first, and then the integral over *g*, instead of using equation (14). Details of the computations are given in Appendix *C* [App appc]. The resulting expression is 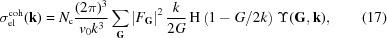
where H(*x*) is the Heaviside step function, which is 0 for *x* < 0 and 1 for *x* > 0, and 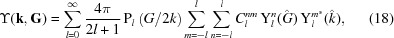
where P_*l*_(*x*) is the Legendre polynomial of order *l*. Again, for a uniform ODF ϒ(**G**, **k**) = 1, and the well known total elastic coherent cross section for the scattering by a powder is recovered (Sears, 1989 ▸). To our knowledge, the above expressions for the total cross section in terms of the Fourier coefficients of the ODF [equations (17) and (18)] have not been derived before, although a similar expression for the angular distribution function is given in the book by Bunge (1993 ▸) when deriving an expression for the inverse pole figure in terms of the series expansion.

Expressions (17) and (18) can be very useful for analysing transmission experiments involving polycrystalline materials. In particular, in neutron imaging experiments with energy resolution, the elastic coherent term contributes to the appearance of the Bragg edges. The position and shape of these edges will depend on the spacing between the diffraction planes of the grains and on the crystallographic texture, respectively. From equation (17), it is clear that the edge for a particular plane **G** starts to contribute to the total cross section when the Heaviside function becomes nonzero, *i.e.* when *G* = 2*k*. Thus, in principle, provided the instrument has sufficient energy resolution, the position of the edge will serve to determine the state of strain of those grains whose reciprocal vector **G** is parallel to the direction of incidence. The shape of the Bragg edge as a function of the incident energy is controlled by the product of three factors: the square of the structure factor, |*F*_**G**_|^2^, the term *k*/2*G* and ϒ given by equation (18). This last term depends on the direction of the incident beam and the scattering plane **G**, and carries all the information regarding the crystallographic texture through the Fourier coefficients, 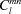. In principle, from a mathematical point of view, the summation over *l*, *m* and *n* in equation (18) prevents the possibility of reconstructing the full ODF of a material from a single transmission experiment, even if it is done with energy resolution. However, it is also clear that, from the combined analysis of a set of transmission experiments with different **k**, some of the 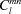 can be approximated by inverting equation (18). This can be useful to obtain from imaging experiments integrated quantities that depend on texture, such as Kearns factors for hexagonal crystals (Kearns, 2001 ▸) or average elastic constants, which only depend on 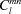 with low *l*.

The total cross section computed from pole figures has been used to analyse transmission experiment data (Santisteban *et al.*, 2006 ▸; Malamud *et al.*, 2014 ▸), but the Fourier expansion presented here may have some advantages. First, for neutron wavelengths of the order of 2 Å or less, several planes contribute to the total cross section, as indicated by the Heaviside function of equation (17). In these cases, integration of the pole figure demands the evaluation of several pole figures, while in equation (17) the contributions of all planes are obtained from the same Fourier coefficients. Second, a good description of the total cross section can be obtained using only a few terms in the case of materials with a soft texture, improving computing times. As a by-product, equation (18) can be used as the basis for ODF inversion problems from transmission experiments. This will be studied in more detail in future work.

Finally, it is worth stressing that both expressions for the differential and total cross section [equations (14) and (17)] are valid for any crystal and sample symmetry. All this information is conveyed by the Fourier coefficients.

## 5. Code implementation   

We have developed code to simulate the scattering of thermal neutrons by a polycrystal using expressions (14) and (17). In order to make it available to the community, we have implemented it in the widely used *McStas* software package (Lefmann & Nielsen, 1999 ▸; Willendrup *et al.*, 2004 ▸, 2019 ▸). The *McStas* component, called Texture.comp, uses the *Union* development of *McStas* (Bertelsen, 2017 ▸), which is very convenient as it allows the separation of physical processes and geometry.

The strategy for simulating the scattering of a neutron of wavevector **k** in a material comprises three steps: (i) sampling the neutron free path to get an interaction point; (ii) sampling the interaction process according to a probability proportional to the scattering cross section of the process; and (iii) sampling the wavevector **k**′ of the outgoing neutron as determined by the differential cross section corresponding to the selected interaction process.

In a homogeneous material, the free path, ξ, is distributed according to an exponential function, 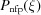 = 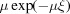, where μ is the linear attenuation coefficient (or macroscopic cross section), given by 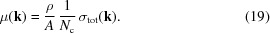
Here, ρ is the density of the material and *A* the mass contained in the crystal unit cell, so that ρ/*A* is the number of unit cells per unit volume, and σ_tot_/*N*_c_ is the total cross section per unit cell. The density can be written as ρ = *pA*/*v*_0_, where *p* ∈ [0, 1] is the packing factor, which can be used instead of ρ/*A*. Thus, for step (i) only μ is required, which, in general, depends on **k**.

The Monte Carlo simulation requires that the polycrystal be statistically homogeneous (*i.e.* homogeneous after averaging over grain disorder). If it is not, it has to be divided into statistically homogeneous pieces. Moreover, only the value of μ averaged over the grain disorder enters the free path distribution, *P*_nfp_(ξ). This is one further approximation that amounts to neglect of the spatial correlations of the grain orientations. Note, however, that this approximation is not specific to the polycrystals with non-uniform texture considered here: it is also used for the simulation of powder samples in the current Monte Carlo codes, although spatial correlations in powders are not expected to be very important.

The linear attenuation coefficient receives additive contributions from the different interaction processes available to the neutron (incoherent elastic, coherent elastic, inelastic *etc.*). Step (ii) selects the interaction process according to the relative probabilities given by the fractional contribution of each process to μ. Thus, for step (ii) only the relative contributions to μ of the available processes are necessary. At step (iii), 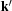 is sampled according to the differential cross section of the interaction process selected at step (ii). The strategy for this sampling depends strongly on the form of the corresponding differential cross section.

In the *Union* development of *McStas*, the geometry and the interaction processes are separated into different components, and multiple scattering is taken into account automatically by the *union master* component, which calls the functions of the components that deal with geometry to perform the ray tracing, and the functions of the components that deal with the interaction processes to sample the free path, the interaction process and 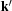. Two functions provide the interface of an interaction process component, such as Texture.comp, with the *McStas* *union master*. One receives **k** as input and returns the contribution of the interaction process to μ. The other one, which is called if and only if the interaction process described by the component is selected by the master at step (ii), again receives **k** as input and returns 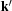.

Let us describe Texture.comp in some detail. It has to compute the functions ϒ and Θ, for which a cut-off, *l*_max_, on *l* has to be used, so that the sum over *l* runs from 0 to *l*_max_. Although the number of terms in the sum is (*l*_max_ + 1)^2^, the computation speed does not depend crucially on *l*_max_, since the main ingredients necessary to obtain Θ and ϒ are precomputed in dense two-dimensional grids of 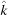 and 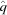 and interpolated as needed in the course of the simulation. This is one of several optimization strategies implemented in the code.

The evaluation of ϒ and Θ is computationally expensive and some strategies to improve the efficiency have been developed. All the terms that do not depend on **k** or **q** can be precomputed and stored in data structures for use in the simulations. The sums entering equations (15) and (18) can be reordered as follows. First we define 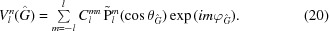
Here, in general 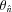 and 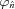 denote the polar coordinates of the unit vector 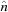 in the sample reference system and 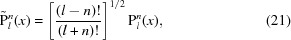
where 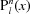 are the associated Legendre functions defined in Appendix *A* [App appa]. It is convenient to work with the polar components of 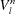, defined by
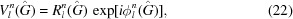
where 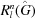 and 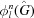 are the modulus and the argument of the complex number 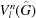, respectively. Then, equations (15) and (18) can be expressed as 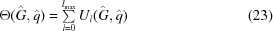
and
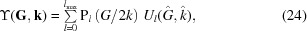
where 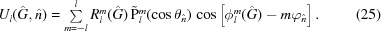
To derive the above equations we have used the fact that the cross sections are real numbers. The vanishing of the imaginary parts of Θ and ϒ can be readily proved using relation (9) and was used as a test for the code, since an expression similar to (25), with 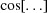 replaced by 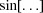, has to vanish.

The computation of Θ and ϒ is too expensive if *l*_max_ is large, even using expressions (23) and (24), with precomputed 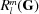 and 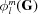. As mentioned above, to speed up the computations 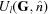 is precomputed, for each **G** and *l*, on a dense two-dimensional grid of 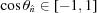 and 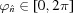. The Legendre polynomials entering (24) are also precomputed on a dense grid of [−1, 1]. Thus, the computation time for ϒ scales as *l*_max_, since it amounts to performing the sum over *l* with the values of the terms obtained by interpolation. In the case of Θ, the whole sum over *l* can be precomputed, and thus its computation time is independent of *l*_max_. That is, 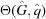 is precomputed, for each **G**, on a dense two-dimensional grid of 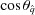 and 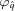.

The contribution to μ of the coherent elastic scattering by the textured polycrystal, denoted here by μ_coh_, is given by μ_coh_ = 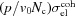, and thus it is numerically computed from equations (17) and (24). The *union master* component uses it to obtain the interaction point and to sample the interaction process. If the coherent scattering by the polycrystal is selected, Texture.comp has to sample the value of the scattered wavevector, 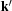. This sampling is explained in what follows.

First, note that, according to equation (17), the probability that the scattering is due to the set of lattice planes perpendicular to **G** is 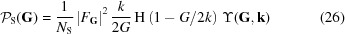
where 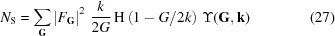
is the normalization factor. The values of 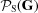 are computed and stored when calculating the contribution of the coherent elastic scattering to μ and need not be computed again. A vector **G** is selected according to the probability 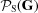. This sampling is standard, since the set of **G** that satisfy the condition *G* < 2*k* imposed by the Heaviside function (the Bragg cut-off) is finite. For the selected **G**, the delta function in equation (14) determines the scattering angle, θ_s_, which is given by 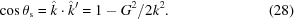
Thus, as is well known, 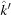 lies on the surface of a cone whose axis is given by 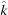 and whose angle is θ_s_ (the Debye–Scherrer cone). It only remains to sample the azimuthal angle, 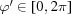, around the cone axis. Introducing two unit vectors 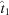 and 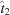 so that 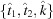 forms a right-handed orthonormal triad, we have 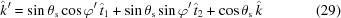
and 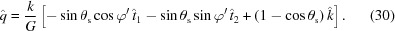
Let us denote by 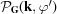 the probability density of 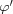. Note that this probability density gives the modulation of intensity of the diffracted beam along the Debye–Scherrer cones. According to equation (14), it is obtained, except for a normalization factor, by substituting the above expression for 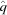 into 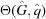, which is computed numerically from equation (23). Hence, given **G** and **k**, the first step is to obtain 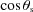, 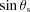, and the two vectors 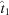 and 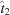. Then, 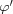 is sampled according to its probability distribution, for which a simple rejection method is convenient. However, rejection methods need an upper bound for the probability density maximum, and their efficiency is worse the higher the upper bound. In our case, global upper bounds can be obtained from equation (23) but, although they work reasonably well in most instances, they are so bad in some cases that the rejection method becomes useless. The solution, although not very efficient, is to compute 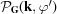 in a sufficiently dense grid in [0, 2π] using equation (23). Its maximum is obtained from the discrete values. This computation has to be performed each time 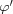 is sampled. The simple rejection method is thus straightforward and works as follows. An angle 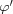 is uniformly selected in [0, 2π], and 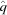 is computed according to equation (30). Then 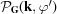 is obtained by linear interpolation on the grid. The ratio of this probability density to the maximum probability density is compared with a random number selected uniformly on [0, 1]. If the ratio is smaller than the random number, the value of 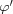 is accepted, 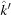 is computed from equation (29) and 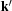 = 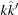. Otherwise, another value of 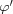 is selected uniformly in [0, 2π] and the process is repeated.

Clearly, the sampling of 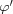 is the bottleneck in the simulation. An improvement in the sampling procedure might dramatically increase the code efficiency. The main problem is that, although we can compute 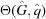 with reasonably efficiency (by interpolating precomputed values), this is not enough. For an importance sampling method, like rejection, we need the maximum in 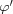 of 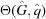, with 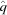 given by equation (30), which depends on the wavevector of the incident neutron, **k**. To estimate the maximum, 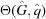 has to be evaluated many times. Alternatively, we might consider using the weight factor transformation,^1^ frequently used in *McStas* (Willendrup *et al.*, 2019 ▸, 2018 ▸). A similar problem arises in this case, however, since the weight is given by the normalized probability density, 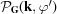, which is proportional to 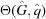, the proportionality factor being the inverse of the integral of 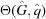 over 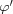. To compute the normalization factor, which again depends on **k**, one has to evaluate 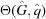 many times. A simple brute-force possibility would be to precompute either the maximum in 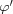 of 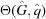 or the normalization factor of 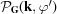 on a three-dimensional grid in **k** space. However, the precomputation would be very time consuming and the three-dimensional table very large, and we prefer to avoid interpolation on three-dimensional grids. Hence, we discarded this possibility. In any case, it is clear that there is ample room for improvement at this point.

The need to compute 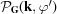 on the 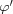 grid each time that 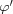 is sampled would make the simulations unfeasible if Θ and ϒ_*l*_ had to be computed from expressions (23) and (25). The precomputation of Θ is crucial. In comparison, the use of the precomputed *U*_*l*_ in the evaluation of ϒ is less important: it greatly improves the efficiency of the simulation, but simulations would still be feasible without it.

The efficiency of the simulation can be greatly improved by using variance reduction techniques (stratified sampling) provided by the *McStas* kernel, especially the use of the SPLIT keyword (Willendrup *et al.*, 2019 ▸, 2018 ▸). Using the SPLIT *n* keyword, each incoming neutron is reused *n* times. The values of μ_coh_ and 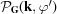 computed on the grid are saved and reused when another identical neutron enters the component.

Summarizing, the user has to provide the *McStas* Texture.comp component with the necessary information through eight input parameters:

(*a*) The paths to three files: one which contains the coordinates of the crystal reference frame, 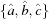, in the sample frame; another one which contains the crystallographic information in Lazy/ICSD format (Yvon *et al.*, 1977 ▸); and a third which contains the Fourier coefficients of the ODF.

(*b*) Four integer numbers: the cut-off *l*_max_; the sizes in each dimension of the 2D grid in 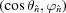 space where 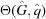 and 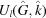 are precomputed, *n*_ct_ × *n*_φ_; and the size of the grid used to sample 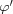, 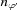.

(*c*) One real number, the packing factor *p*.

The program obtains the Miller indices and the corresponding structure factors from the crystallographic file. The user has to guarantee consistency between the sample frame, the crystal reference frame, the crystallographic information and the Fourier coefficients of the ODF.

## 6. Cut-off effects   

**Figure 1:**
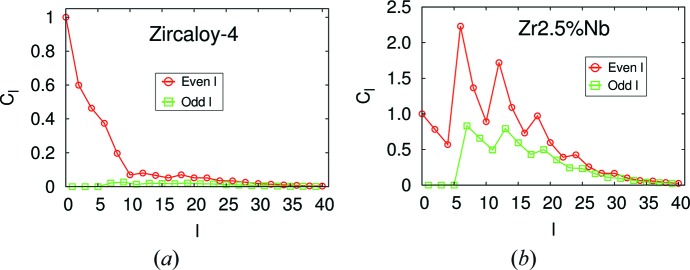
Fourier coefficients of (*a*) the Zircaloy-4 plate ODF and (*b*) the Zr–2.5Nb pressure tube ODF. What is actually plotted is 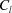 = 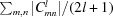.

The feasibility of Monte Carlo simulations using the method proposed in this work relies on the truncation of the expansion, restricting the sum in *l* to *l* ≤ *l*_max_, with *l*_max_ sufficiently small. To investigate the cut-off effects, we considered the textures of a Zircaloy-4 plate and a Zr–2.5Nb pressure tube that were obtained experimentally by Malamud *et al.* (2018 ▸). The coefficients 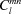 are computed from the ODFs reported in this reference, using the *MTEX* software. Fig. 1 ▸ displays 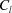 = 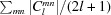 as a function of *l* for the two materials. The Zr–2.5Nb pressure tube has a sharper texture than the Zircaloy-4 plate, which is reflected in the slower vanishing of the Fourier coefficients as *l* → ∞.

The dependence on *l*_max_ of the pole figures 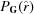 associated with the lattice planes perpendicular to the reciprocal-lattice vector **G** is obtained by truncating at *l*_max_ the sum in *l* in the well known relation (Bunge, 1993 ▸) 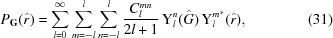
where 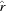 represents a direction relative to the sample reference frame.

**Figure 2:**
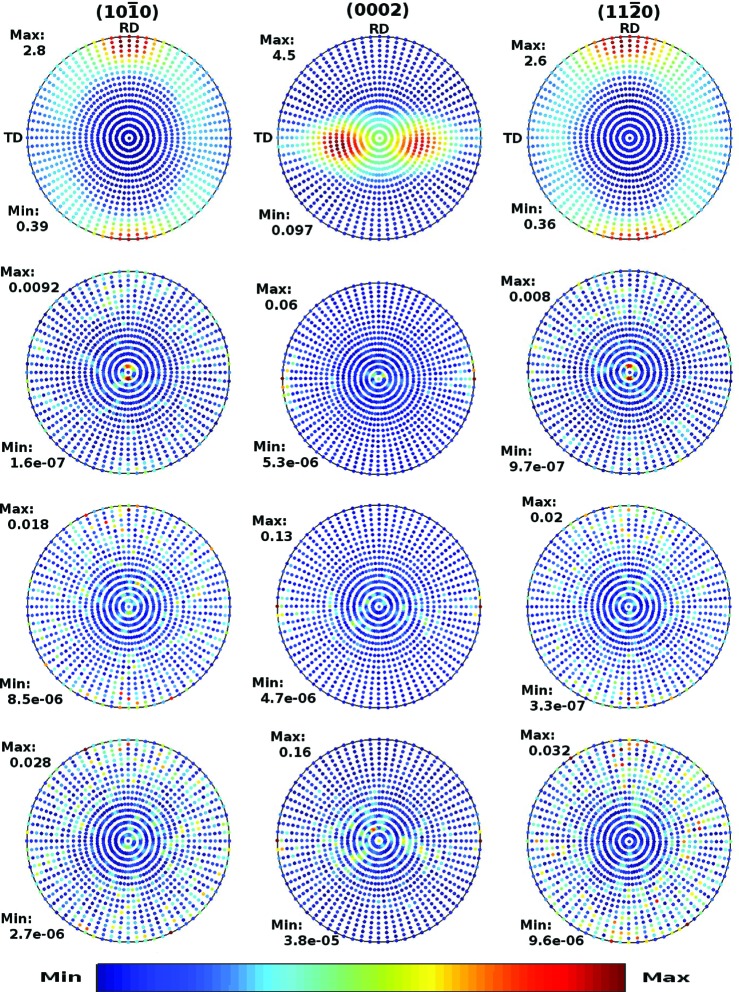
Pole figures of the Zircaloy-4 plate corresponding to the crystal planes (left) 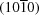, (middle) (0002) and (right) 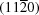. In the first row they are computed from the experimental ODF of Malamud *et al.* (2018 ▸). The second, third and fourth rows display the differences between the pole figures shown in the first row and those computed using equation (31) with cut-off *l*_max_ = 30, 25 and 20, respectively, and Fourier coefficients obtained from the ODF of Malamud *et al.* (2018 ▸).

**Figure 4:**
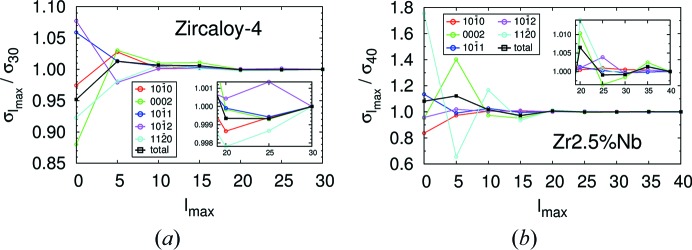
The contributions of different crystallographic planes to the total cross section of (*a*) the Zircaloy-4 plate and (*b*) the Zr–2.5Nb pressure tube, as a function of the cut-off *l*_max_. To appreciate the convergence to the *l*_max_ → ∞ limit, they are normalized to the value at the largest *l*_max_.

The cut-off effects on pole figures in the Zircaloy-4 plate and Zr–2.5Nb pressure tube are displayed in Figs. 2 ▸ and 3 ▸, respectively. The columns, from left to right, correspond to the 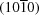, (0002) and 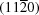 crystal planes, respectively. The top panels display the pole figures computed directly from the experimental ODF [*cf.* Figs. 4 and 6 of Malamud *et al.* (2018 ▸)]. The lower panels display the absolute difference between the pole figures shown in the top panels and the pole figures computed using equation (31), with cut-offs *l*_max_ of 30, 35 and 20 in the Zircaloy-4 case, and of 40, 35 and 30 in the Zr–2.5Nb pressure tube case. The pointwise convergence of the Fourier expansion can be appreciated by looking at the scale set by ‘Max’ in the figures.

The dependence of the cross sections on *l*_max_ is also interesting. Fig. 4 ▸ displays, as a function of *l*_max_, the contribution of different *hkl* planes to the total scattering cross section for a neutron of λ = 3.1 Å propagating along a direction given by polar and azimuthal angles of 80 and 60°, respectively, with respect to the sample reference frame. These angles were chosen arbitrarily and correspond to an impinging direction 80° to the normal direction and 60° to the rolling direction in the case of the Zircaloy-4 plate, and 80° to the radial direction and 60° to the axial direction in the case of the Zr–2.5Nb tube. The left- and right-hand panels correspond to the Zircaloy-4 plate and the Zr–2.5Nb pressure tube, respectively. The total cross section is also displayed (black line). To appreciate the convergence towards the *l*_max_ → ∞ limit, the values are normalized by those with the largest cut-off (30 and 40 for the Zircaloy-4 plate and the Zr–2.5Nb pressure tube, respectively). The insets magnify the region of larger *l*_max_. We see that the uncertainties introduced by the finite value of *l*_max_ are very small if *l*_max_ is large enough.

**Figure 5:**
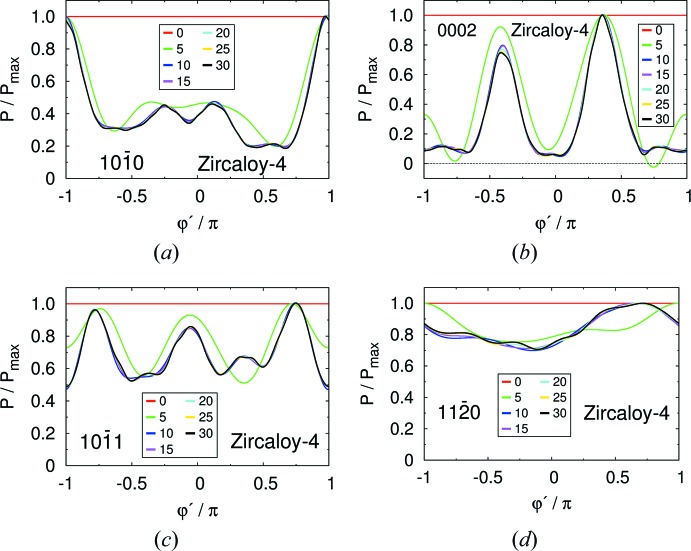
The probability density, 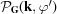, of 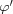 for the Zircaloy-4 plate for different crystallographic planes and a fixed **k** (see main text), for the values of *l*_max_ displayed in the legends.

Fig. 5 ▸ displays the probability density, 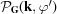, of 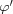 for the scattering of a neutron with wavevector **k** described in the preceding paragraph by various crystal planes of the Zircaloy-4 plate. The probability density is normalized by its maximum. Remember that this function corresponds to the modulations of intensity around the corresponding Debye–Scherrer cone diffracted by a small sample. Each panel corresponds to a different set of crystal planes, whose Miller indices are shown. The different curves correspond to different values of *l*_max_, displayed in the legend. For *l* = 0, the curves are constant, as they have to be for a uniform texture, with no preferred orientation. Note that the differences decrease considerably as *l*_max_ is increased and are large only for *l*_max_ < 10.

**Figure 6:**
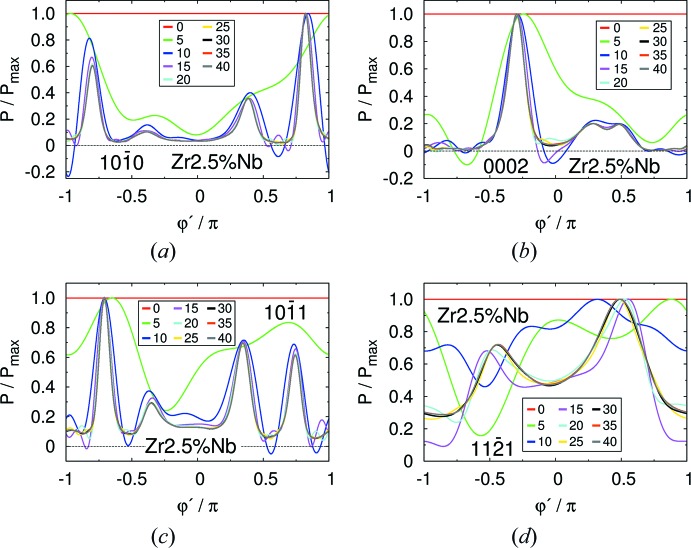
The same as Fig. 5 ▸ but for the Zr–2.5Nb pressure tube.

The analogous data for the Zr–2.5Nb pressure tube are displayed in Fig. 6 ▸. While for the Zircaloy-4 plate the curves converge for *l* > 30, for the Zr–2.5Nb texture convergence occurs for *l* > 40. This is consistent with the higher texture sharpness of the Zr–2.5Nb tube, as discussed by Malamud *et al.* (2018 ▸) and observed in Figs. 2 ▸ and 3 ▸. In the Zr–2.5Nb case there are appreciable differences for *l*_max_ ≤ 15, but for *l*_max_ ≥ 20 the differences are small. Note that for *l*_max_ ≤ 15 the probability density even becomes negative in some regions. In these regions, however, the true probability is small. The code deals with this problem just by replacing the negative probabilities by zero. This is reasonable since, for instance, some useful although not accurate estimation of the background generated by a Zr–2.5Nb pressure tube might be obtained by Monte Carlo simulations using *l*_max_ = 15. Nevertheless, it is advisable to increase the value of *l*_max_ since, due to the optimization implemented in the code, this will not significantly affect the efficiency of the simulations.

## 7. Code validation   

To validate the code we performed Monte Carlo simulations to obtain the pole figures of the Zircaloy-4 plate and the Zr–2.5Nb pressure tube, and compared the results with the exact pole figures displayed in the upper panels of Figs. 2 ▸ and 3 ▸. By exact we mean that these are the pole figures that correspond to the set of Fourier coefficients used in this work, which obviously suffer from the uncertainties associated with their experimental and theoretical determination. The Monte Carlo simulations were performed with the same Fourier coefficients and cut-off, and therefore have to reproduce them with high accuracy. All simulations described in this paper (in this and the next section) were performed by precomputing 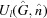 and 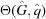 on a 201 × 601 uniform grid in 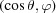 space. The grid could probably be made coarser with no great loss of accuracy, but we have not systematically studied the trade-off between simulation accuracy and grid density.

**Figure 7:**
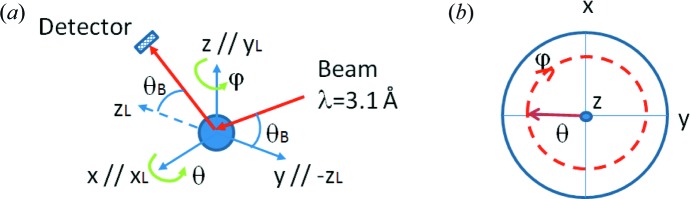
(*a*) A scheme of the experimental setup used for the simulation of the pole figure measurement. The sample coordinates correspond to (*x*, *y*, *z*) = (RD, TD, ND) for the Zircaloy-4 plate and (*x*, *y*, *z*) = (AD, HD, RD) for the Zr–2.5Nb pressure tube. (*b*) A pole figure scan with the experimental setup shown in panel (*a*).

To avoid systematic uncertainties, the simulations are performed in an almost ideal case: the beam, highly collimated (with negligible divergence) and perfectly monochromatic, with λ = 3.1 Å, is scattered by a small spherical sample of 1 mm radius, and multiple scattering is forbidden. The area of the detector is chosen to be small enough that its influence on the results is negligible. The statistical uncertainties are kept low by simulating a high number of neutron histories. Another source of uncertainty is introduced by the approximations made in the code to optimize the computations (for instance, interpolations of precomputed quantities). Although not convenient in real neutron experiments, in this virtual experiment the Schulz setup (Schulz, 1949 ▸) is used, as shown in Fig. 7 ▸(*a*): the direction of the incident beam and the position of the detector are chosen so that the scattering vector is always directed along the 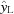 direction in the laboratory reference frame, given by the orthonormal triad 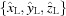. The sample is initially positioned so that 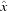 = 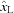, 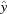 = 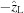 and 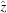 = 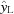. The vectors of the sample reference frame, 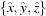, are identified with, respectively, the rolling direction (RD), the transverse direction (TD) and the normal direction (ND) in the Zircaloy-4 plate case, and with the axial direction (AD), the hoop direction (HD) and the radial direction (RD) in the Zr–2.5Nb pressure tube case. Then, the sample is rotated by an angle θ about 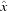 and subsequently by an angle φ about 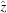, and a Monte Carlo simulation is performed. This process is repeated in steps of 5° in both θ and φ, starting from θ = 0 and φ = 0. Fig. 7 ▸(*b*) displays the projection of the scattering vector onto the pole figure as the two rotations on θ and φ are performed. It is clear that, with the set of rotations proposed, a full coverage of the pole figure is achieved.

**Figure 8:**
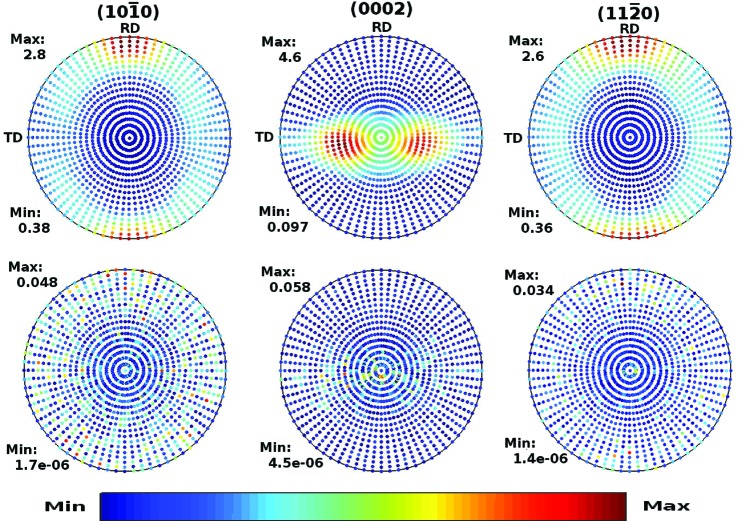
Pole figures of the Zircaloy-4 plate for the crystal planes (left) 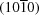, (middle) (0002) and (right) 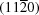 from a Monte Carlo simulation with *l*_max_ = 20. The bottom panels display the differences relative to the exact result given by equation (31).

**Figure 10:**
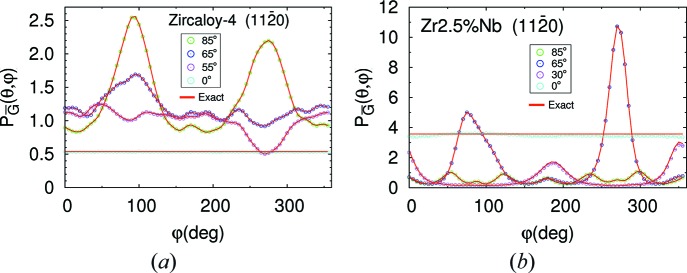
Cuts at constant θ of the 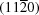 pole figure of (*a*) a Zircaloy-4 plate computed with *l*_max_ = 20 and (*b*) a Zr–2.5Nb pressure tube with *l*_max_ = 30. The values of θ are displayed in the legends. The points are the results of a Monte Carlo simulation and the continuous red line the exact result obtained from equation (31) with the corresponding cut-off.

The pole figures obtained from the Monte Carlo simulation are displayed in Figs. 8 ▸ and 9 ▸ for the Zircaloy-4 with *l*_max_ = 20 and for the Zr–2.5Nb pressure tube with *l*_max_ = 30, respectively. The lower panels show the absolute differences relative to the exact result; the scale of the figures indicates that they are small. To appreciate better the quality of the simulations, Fig. 10 ▸ displays several cuts at constant θ of the 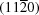 pole figure. These curves represent the variation in intensity in the pole figure along the circle centred at *z* with radius θ, as shown in Fig. 7 ▸(*b*). The symbols are the results of a high-statistics Monte Carlo simulation and the continuous red line is the exact result, obtained with the appropriate truncation of equation (31). The error bars signalling the statistical uncertainties of the simulations, smaller than the symbols, are barely visible. The left- and right-hand panels correspond to, respectively, the Zircaloy-4 plate, with *l*_max_ = 20, and the Zr–2.5Nb pressure tube, with *l*_max_ = 30.

We also performed simulations to validate the implementation of the linear attenuation coefficient. This implementation, however, is much easier than the implementation of the scattering process, which, as described in Section 5, has to sample 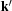 according to the proper probability distribution. For the linear attenuation coefficient we only had to implement in the *McStas* code the computation of μ_coh_ using equations (17) and (18). The *McStas* *union master*, which has been validated elsewhere (Bertelsen, 2017 ▸), takes care of sampling the interaction point and the interaction process.

**Figure 11:**
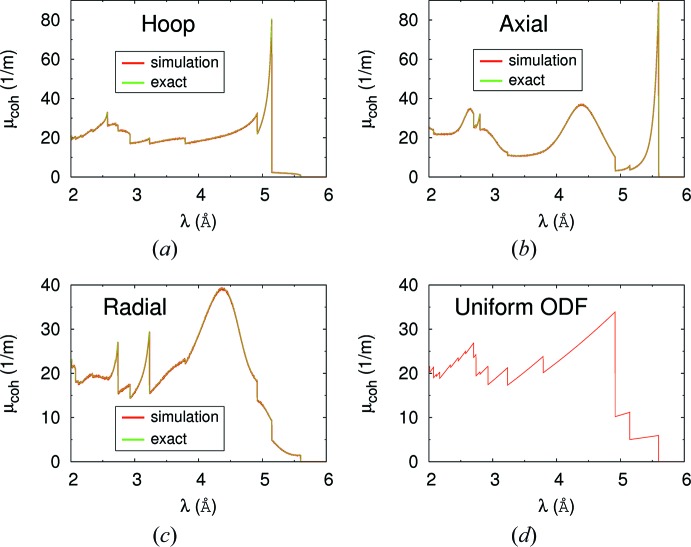
Simulation of neutron transmission through a small sample under ideal conditions. The upper panels and the bottom left-hand panel correspond to a Zr sample with the texture of the Zr–2.5Nb pressure tube reported by Malamud *et al.* (2018 ▸). In each case the beam propagates along the sample direction displayed in the figure. The bottom right-hand panel corresponds to a Zr sample with uniform ODF. The red lines are the results of the simulation and the green lines the exact result. In the first three panels the tiny differences are due to noise in the simulation results. In the last panel (bottom right) the differences between the simulation and the exact result cannot be appreciated on the scale of the figure.

The simulations setup is as follows. A very small rectangular sample, with dimensions 0.1 × 0.1 × 1 mm, is irradiated with an almost perfectly collimated beam, with divergence smaller than 1.0 × 10^−4^ °, and with a uniformly distributed wavelength, λ, between 2 and 6 Å. Two detectors with wavelength resolution are placed in front of and behind the sample. To avoid uncertainties we force *McStas* to absorb neutrons that suffer interaction. In this way, the detector behind the sample collects the neutrons that traverse the sample without interaction, while the detector in front of the sample merely counts the number of incident neutrons. If *I*_0_(λ) and *I*_1_(λ) are the intensities recorded by the detectors in front of and behind the sample, respectively, the simulated linear attenuation coefficient is given by 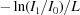, where *L* = 1 mm is the transmitted neutron path length through the sample. The exact value is computed independently from equations (17) and (18) without relying on *McStas*. Fig. 11 ▸ displays the results. The red and green lines are, respectively, the results of the simulation and the exact values. The panels, from left to right and from top to bottom, correspond to, respectively, beams propagating along the hoop, the axial and the radial directions through a sample with the texture of the Zr–2.5Nb pressure tube, and along the *Z* direction through a Zr sample with uniform ODF. Note the perfect agreement (within the simulation noise) between the simulation and the exact results.

Note also the large difference between the attenuation coefficient of a textured material and another with a uniform ODF. The results displayed in Fig. 11 ▸ are similar to those presented in Fig. 8 of Santisteban *et al.* (2012 ▸). In that work, experimental values for a Zr–2.5Nb pressure tube with similar texture to the one considered in this work were compared with theoretical evaluations obtained using the technique of integrating pole figures to compute the total coherent elastic cross section.

The perfect agreement between the Monte Carlo results and the exact pole figures and attenuation coefficients is a strong indication that the code works properly. In the case of the Zr–2.5Nb pole figures, very small but sizable (larger than three standard deviations of the statistical uncertainty) discrepancies between the Monte Carlo results and the exact values are evident for θ = 0° and θ = 30°. They are caused by the unavoidable systematic effects of the simulation such as, for instance, the size of the detector, which is small but finite, and the systematic approximations made in the algorithm (*e.g.* the interpolation of precomputed values and the finite size of the grids).

## 8. Example: signal-to-noise ratio in a simplified model of a pressure cell   

As an example, we simulated the SNR in a simple experiment in which a powder sample of Na_2_Ca_3_Al_2_F_14_ (Courbion & Ferey, 1988 ▸) is located inside a cylindrical container that simulates a pressure cell, with a wall made of Zr with the Zr–2.5Nb texture.

Note that, generically, the separation of the detector readings into signal and noise components is not universally defined, but depends on the experimental goals: what in one experiment is part of the signal might be considered noise in a different experiment. Since we are not concerned here with a particular experiment, but with the background generated by the instrument, we consider as signal all neutrons scattered only by the sample, and as noise neutrons scattered at least once by the container. Hence, the SNR is defined here as the ratio between the number of neutrons that reach the detectors after having been scattered only by the sample (one or more times) and the number of neutrons that reach the detectors after having been scattered at least once by the container (and perhaps also by the sample). In some experiments, however, neutrons scattered by the sample incoherently or more than once would be considered noise generated by the sample.

**Figure 12:**
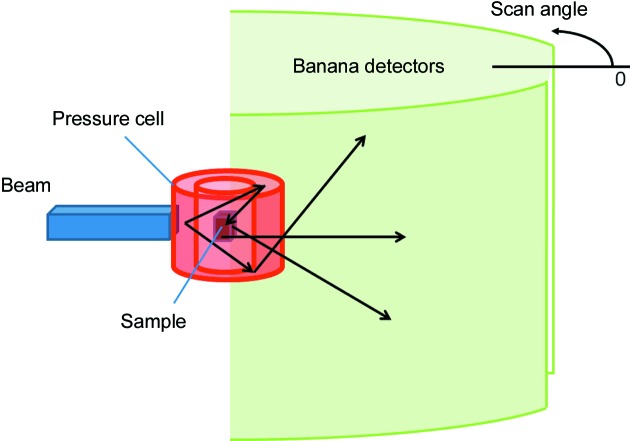
A scheme of the simulated experiment to estimate the SNR. A cylindrical sample is located inside a hollow cylinder modelling a pressure cell made of a Zr alloy. Two detectors of cylindrical shape are placed to have almost 2π coverage (only parts of them are shown). Neutrons (black lines) may suffer from multiple scattering by the different components before reaching the detector.

Fig. 12 ▸ displays the setup. The container is a hollow cylinder with a diameter of 18 mm, height of 150 mm and thickness of 3 mm, so that inside there is an empty cylindrical space of 12 mm in diameter. The sample has cylindrical geometry, 6 mm in diameter and 10 mm in height. The system is irradiated with a neutron beam that has a Gaussian-distributed wavelength of 3 ± 0.015 Å and a Gaussian distribution of divergence with a standard deviation of 0.4°. The beam is limited by a 6.6 × 110 mm slit, a bit larger than the sample, located 30 cm before it. The scattered neutrons are collected by two area detectors with the geometry of cylindrical sectors of 1 m radius, centred at the sample position, which are 4 m high (vertical direction) and cover angular intervals with respect to the beam direction from 5 to 170° and from 190 to 355°, respectively. By symmetry, the intensity collected by both detectors is essentially the same, and we only show the results for the first detector.

The texture of the Zr–2.5Nb pressure tube is actually the texture of a small cylindrical sector cut from the tube. That means a whole cylinder is composed of many small cylindrical sectors, with the texture of each sector oriented according to the corresponding AD, HD and RD directions. Hence, we cannot simply simulate a whole cylinder with the same texture, relative to the laboratory frame, at any point. Rather, we have to divide the cylinder into small sectors and assign to each sector the texture oriented according to the local AD, HD and RD directions. In practice we divide the cylinder into 24 sectors of 15°, which causes a big increase in the simulation complexity.

**Figure 13:**
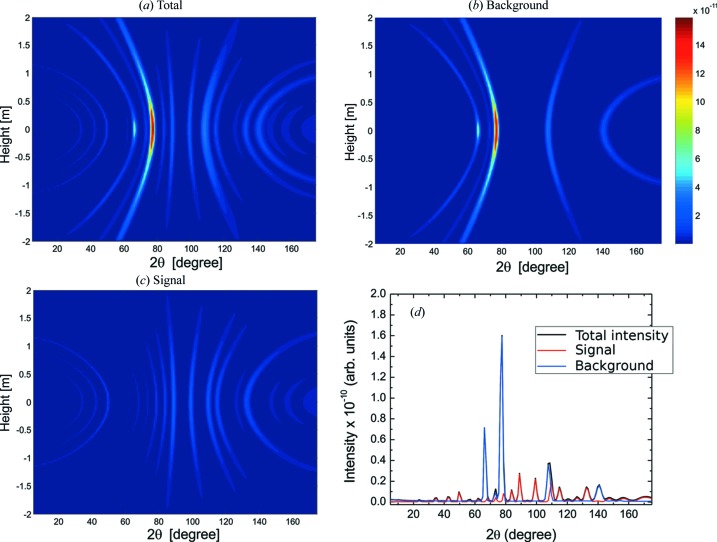
The intensity collected by the detectors in the simulation of an experiment with the simplified model of a pressure cell described in Section 8, with the cell walls made of Zr with the texture of the Zr–2.5Nb pressure tube reported by Malamud *et al.* (2018 ▸). (*a*) Total intensity, (*b*) intensity of neutrons scattered at least once by the cell walls (background), (*c*) intensity of neutrons scattered only by the sample (signal) and (*d*) intensity of neutrons collected by the detectors in the equatorial plane, providing a typical diffractogram: total (black), signal (red) and background (blue).

Fig. 13 ▸ displays the results. Panel (*a*) shows the total neutron intensity, in arbitrary units, collected by the detectors; panel (*b*) displays the intensity of the background, *i.e.* of neutrons that have been scattered at least once by the container (and some of them also by the sample); panel (*c*) displays the signal, *i.e.* the intensity of neutrons that have been scattered only by the sample, showing the intersection with the detectors of the corresponding Debye–Scherrer cones; and panel (*d*) displays the total intensity and its components, signal and background, along the equatorial plane of the detector system, as a function of the angular position. This provides a typical diffractogram. Note that at some points the background is much higher than the signal. The lines seen in panel (*b*) correspond to the intersection of the detector surface with Debye–Scherrer cones originated by the wall material, which is not at the centre of the detector system. Therefore, several factors contribute to the modulations along the rings: the crystallographic texture, the differences in neutron path length through the various materials, which causes differences in attenuation, and the differences between the solid angles subtended by the detectors and interaction points.

**Figure 14:**
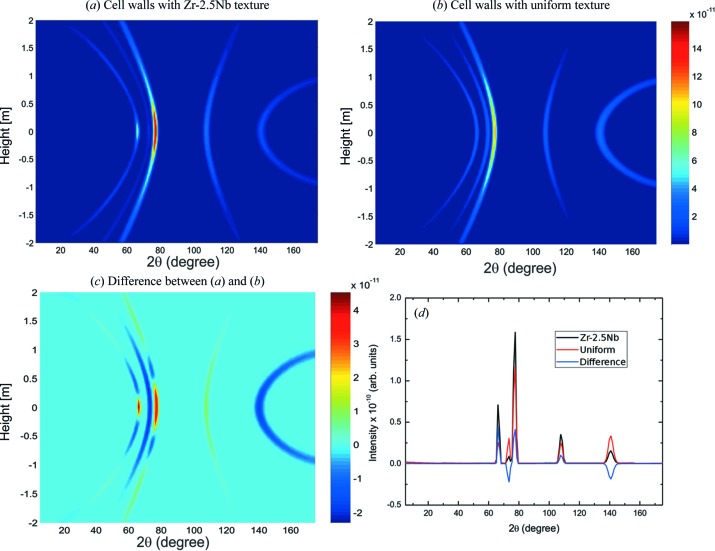
The intensity collected at the detectors of neutrons scattered only by the cell walls, (*a*) with the texture of the Zr–2.5Nb pressure tubes and (*b*) with a uniform texture. The difference is displayed in (*c*) and the results in the equatorial plane in (*d*).

To analyse the effect of the cell wall texture, the simulation has been repeated considering a Zr wall with uniform ODF. The results are displayed in Fig. 14 ▸. Panels (*a*) and (*b*) show the intensity of neutrons scattered only by the cell wall, with the Zr–2.5Nb texture and with the uniform ODF, respectively. Fig. 14 ▸(*a*) is essentially indistinguishable from Fig. 13 ▸(*b*), which means that the intensity of neutrons scattered both by the cell walls and by the sample is small. The different modulations of the intensity along the Debye–Scherrer cones in panels (*a*) and (*b*) of Fig. 14 ▸ are due to the texture, which has a big influence on the background, as seen in panel (*c*), where the difference between the intensities of neutrons scattered by both types of cell wall is displayed. The results in the equatorial plane, as a function of the angular position, are shown in panel (*d*).

**Figure 15:**
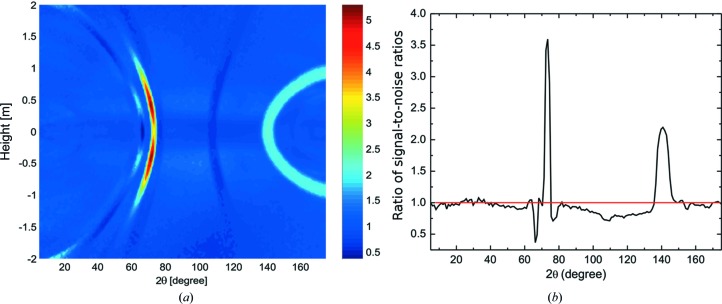
(*a*) The ratio of SNR_1_/SNR_2_ of the SNR of a pressure cell with Zr walls with the texture of the Zr–2.5Nb pressure tubes (SNR_1_) and with a uniform texture (SNR_2_). (*b*) A cut of panel (*a*) along the equatorial plane of the detector system, height = 0.

The texture has an important influence on the SNR, as expected. The left-hand panel of Fig. 15 ▸ displays the ratio SNR_1_/SNR_2_, where SNR_1_ and SNR_2_ stand for the SNR of a Zr pressure cell with the Zr–2.5Nb texture and with uniform ODF, respectively. The right-hand panel shows the data along the equatorial plane of the detector system. There are points at which the SNRs differ by a factor higher than five. Note that, although the SNR depends crucially on the sample, which provides the signal, the ratio of SNRs in the case of two different containers is nearly independent of the sample, since the influence of the container on the signal is rather small. Thus, the ratio of SNRs is, to high accuracy, the ratio of the background produced by the containers, which, in turn, depends very little on the sample. This means that Fig. 15 ▸(*a*) can be visualized, to a very good approximation, as the point-to-point ratio of Figs. 14 ▸(*b*) and 14 ▸(*a*).

## 9. Summary and conclusions   

We have developed a method of simulating the transport of thermal neutrons through polycrystalline materials. It is based on the generalized Fourier expansion, in terms of Wigner *D* matrices, of the orientation distribution function, which leads to an expansion of the differential and total cross sections in terms of the Fourier coefficients. These expansions are suitable for Monte Carlo codes. As expected, the expression for the differential cross section associated with a crystal plane is proportional to the well known analogous expansion of the corresponding pole figure (Bunge, 1993 ▸). Although alternative expressions are currently used, to our knowledge the expression for the total cross section given here has not been derived before. In some cases, for instance in Monte Carlo codes, it has advantages over other expressions.

The method has been implemented in a *McStas* component code called Texture.comp. It has been validated by computing the pole figures of a Zircaloy-4 plate and a Zr–2.5Nb pressure tube through Monte Carlo simulations of an ideal neutron diffraction experiment, where the sample is rotated about two axes, and by simulating a transmission experiment under ideal conditions. As a first application, we estimated the signal-to-noise ratio of a diffraction experiment in which a small sample is placed inside a cylindrical pressure cell made of a Zr alloy with the texture of the Zr–2.5Nb pressure tube obtained by Malamud *et al.* (2018 ▸). To see the effect of texture, the simulation was repeated considering a Zr alloy with uniform texture. We found that texture has a deep impact on the SNR: at some points the two SNRs differ by a factor greater than five.

The computational cost of simulating thermal neutron transport through textured polycrystals is obviously much higher than that through a polycrystal with a uniform ODF. The cost depends strongly on the complexity of the problem. The higher the complexity, the higher the relative cost of the problem with non-trivial texture. For the simplest problem, in which neutrons are scattered only by a small sample, so that multiple scattering is very unlikely, the computing time in the non-trivial texture case is only three and a half times longer than that in the uniform ODF case, if the *McStas* SPLIT keyword is used heavily. Without using SPLIT, it is 12 times longer. For complex problems the SPLIT keyword is not as effective. For instance, in the problem described in Section 8, which is rather complex since the cylindrical container with non-trivial texture was divided into 24 sectors, the simulations (using SPLIT) with the Zr–2.5Nb texture were 32 times longer than with the uniform ODF. This is, however, not a big problem, given (i) the power of current computation resources, (ii) that the simulations are trivially parallelizable and (iii) that *McStas* is extremely fast at simulating powder materials.

The generalized Fourier expansion of the ODF is useful only if the texture is sufficiently mild. For very sharp textures, the ODF can be split into a smooth component and some sharp peaks. The smooth part can be simulated with the software presented here and the sharp peaks with the methods proposed by Laliena *et al.* (2019 ▸).

The software will be used to compute the background generated by components like pressure cells in neutron scattering instruments, which depends strongly on the texture of the device materials. The final goal is to assist in the design of neutron instruments for extreme conditions by estimating, through Monte Carlo simulations, the SNR in different configurations. The software, however, has a much broader scope, and may also be used for the analysis of experiments involving samples of polycrystalline materials, like pole figures and residual stresses in alloys. Interestingly, the expression for the total cross section can be used to analyse data from transmission experiments with polycrystalline materials (Vicente-Álvarez *et al.*, work in progress).

The component Texture.comp will appear in the next *McStas* release, so that it will be available to the community. It can also be obtained in advance from the authors upon request.

## Figures and Tables

**Figure 3:**
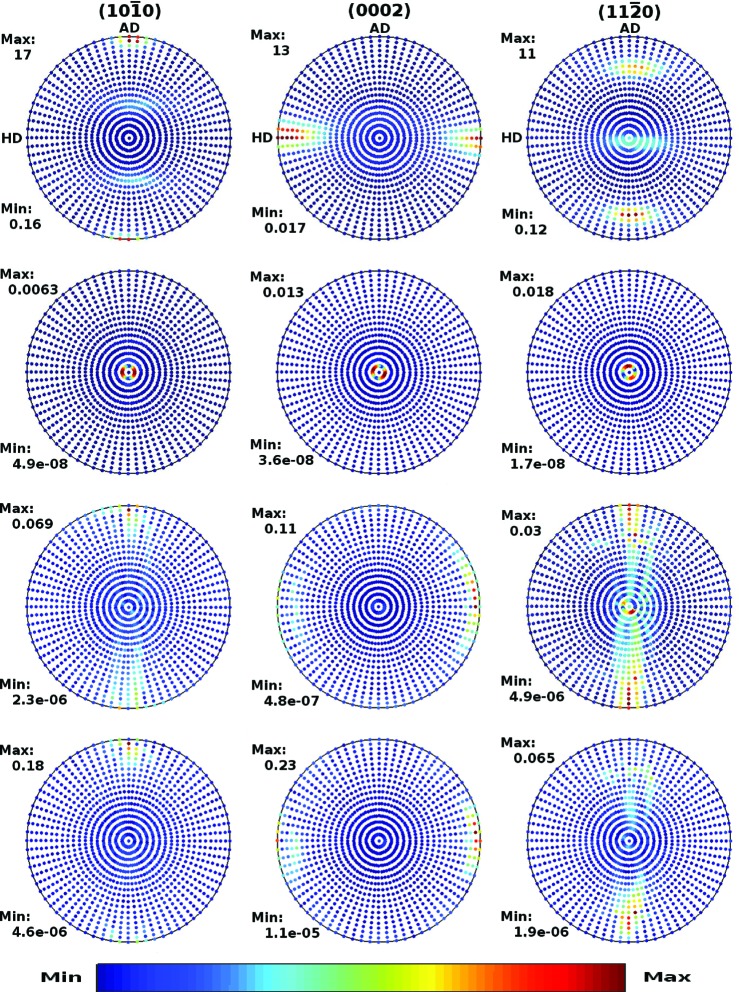
The same as Fig. 2 ▸ but for the Zr–2.5Nb pressure tube. The second, third and fourth rows correspond to *l*_max_ = 40, 35 and 30, respectively.

**Figure 9:**
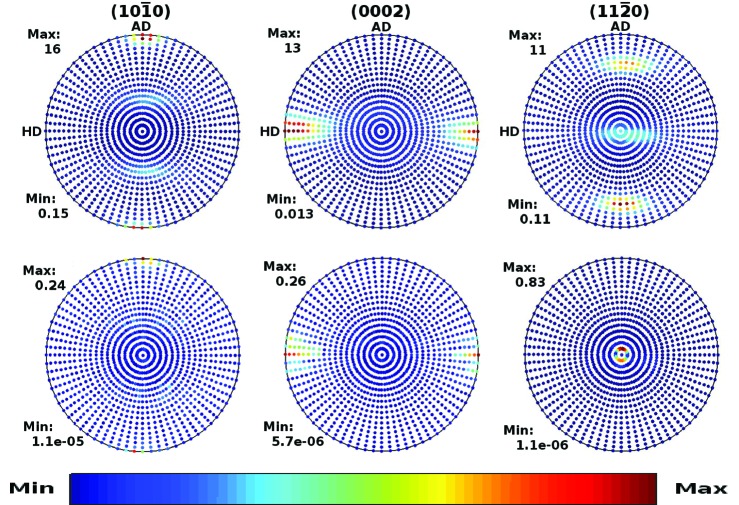
The same as Fig. 8 ▸ but for the Zr–2.5Nb pressure tube with *l*_max_ = 30.

## Footnotes

1 We are grateful to one of the referees for bringing the weight factor transformation to our attention. It might have some advantages over importance sampling methods, opening a way for improving the code efficiency, although the problems discussed in the text have to be overcome first.

## Appendix A Some properties of SO(3)

The Hermitian infinitesimal generators of SO(3) satisfy the algebra

The irreducible representations of SO(3) are characterized by the non-negative integers *l* associated with the eigenvalues, *l*(*l* + 1), of  = , and have dimension 2*l* + 1. An orthonormal basis of the representation space is given by the eigenvectors  of *L* ^2^ and *L*_*z*_, with the integer *m* bounded by −*l* ≤ *m* ≤ *l*. This basis is uniquely determined by choosing a phase convention. Here we adhere to the Condon & Shortley (1957 ▸) choice of phases, which is fixed by the relation

where .

For each *g* ∈ SO(3), the Wigner *D* matrix is defined by

where *R*_*l*_(*g*) is the operator that implements the action of *g* in the *l* representation, which implies the relation

In addition, the unitarity of the representation implies that the Wigner matrices are unitary and

In terms of Euler angles the Wigner matrices read

so that

where

is called the Wigner *d* function. We have the following explicit formula (Galindo & Pascual, 1990 ▸):

Below we will use the following relation (Galindo & Pascual, 1990 ▸):

where  are the spherical harmonics, defined by

with 0 ≤ θ ≤ π and 0 ≤ φ < 2π, and  are the Legendre associated functions, given by

Note that the Legendre polynomial P_*l*_(*x*) of order *l* is the associated Legendre function of order *l* and *m* = 0. The arguments of the spherical harmonics define a unit vector, , given by the polar angle θ and the azimuthal angle φ, so that we can use the notation . Other useful properties of the Wigner matrices and the representations of SO(3) can be found in many books, for instance in Appendix *B* of Galindo & Pascual (1990 ▸).

## Appendix B Computation of the differential cross section

To compute the integral *I*_**G**_(**q**) of equation (13) we use the representation of the three-dimensional Dirac delta function in polar coordinates:

where  is the Dirac delta function on the sphere, which in polar coordinates θ and φ reads

and δ_P_(φ) is the periodic Dirac delta function. Then, introducing the Fourier representation of the ODF, *f*(*g*), we have

where

with . Let  denote a rotation that brings a unit vector  to , so that . Since the Dirac delta function on the sphere is rotationally invariant, we have

Using the invariance of the measure we have

Since the Wigner *D* functions support a unitary representation of SO(3) [equation (35)], we have

The integral can be readily evaluated in terms of the Euler angles, (α, β, γ), that characterize *g*, since

so that

Taking into account that , we obtain

and therefore

The Euler angles corresponding to a rotation  that brings  to  are α = 0,  and , where  and  are the polar coordinates of , and therefore, using relation (41), we have

Using the above expression, equation (54) becomes

and inserting this expression into equation (46) we obtain the desired result.

## Appendix C Computation of the total cross section

The total cross section can be obtained by integrating over the solid angle  the differential cross section given by equation (14). However, it is easier to integrate equation (12) over  before performing the integral over d*g*. Since the scattering is elastic, we have , so that

The integral over  of the above expression is 1, since the first Dirac delta function forces  to lie on the unit sphere. Therefore we have

Let us remember that we defined  as the rotation that brings the unit vector  to , so that . Then we have

Using the invariance of the measure we can write

and, using the Fourier expansion of the ODF and the group properties of the Wigner matrices, we have

where

Using equation (51) we have

which is independent of α and γ. Therefore, the integrals over these angles give . Making the change of variable *z* =  and taking into account that  = , we have

The argument of the delta function vanishes for *z* = *G*/2*k*, and its derivative at this point is −*G*. Then we have

Inserting the above result into (61) and using equation (55) we get
